# Does temporary location of ambulances (“fluid deployment”) affect response times and patient outcome?

**DOI:** 10.1186/s12245-015-0084-1

**Published:** 2015-10-09

**Authors:** Mahmoudreza Peyravi, Soheila Khodakarim, Per Örtenwall, Amir Khorram-Manesh

**Affiliations:** Pre-hospital and Disaster Medicine Centre, Department of surgery, Institute of clinical sciences, Sahlgrenska Academy, Gothenburg University, Regionens HUS SE-40544, Gothenburg, Sweden; Faculty of Shiraz University of Medical Sciences, Shiraz, IR Iran; Departments of Epidemiology, Faculty of Public Health, Shahid Beheshti University of Medical Sciences, Tehran, Iran

**Keywords:** Emergency medical services, Response time, Mortality, Temporarily stationed, Ambulances

## Abstract

**Background:**

The objective of this paper is to evaluate the response times and outcome of patients in two groups of patients attended by permanently (PS) and temporarily stationed ambulances (TS) (fluid deployment).

**Methods:**

Patients transported and treated by EMS between March 21, 2012 and March 20, 2013 in a city with 1.7 million inhabitants (Shiraz, Iran) were studied. Using the same number of ambulances, patients were divided into two groups: transported by ambulances dispatched from permanent ambulance stations (PS) vs. dispatched from temporary locations (TS). Furthermore, due to a high discrepancy in the number of missions between PS and TS in this group, a pilot study was also conducted to confirm the first result. The results were statistically analyzed using various methods and compared with regard to mortality and response time.

**Results:**

In this study (both periods), ambulances dispatched from TS had a reduction of their mean response times by 2 min compare to ambulances dispatched from PS. The difference was statistically significant (*p* < 0.001–[95 % CI, 1.975, 2.025]). The pre-hospital mortality rate was also significantly lower for this group (*p* = 0.04–[95 % CI, 0.006, 0.012]).

**Conclusions:**

The results of this study suggest that temporary deployment of ambulances reduce response times and may improve early survival rates in patients managed by EMS.

## Background

A review of emergency medical systems’ (EMS) activities in low and middle income countries (LMIN) reveals that EMS have difficulties in providing acceptable care. One reason is inadequate investments in infrastructure (vehicles and staff) [1–4], causing public distrust with the EMS and thus enhancing other means of transportation of patients to healthcare facilities [2, 5, 6].

**Fig. 1:**
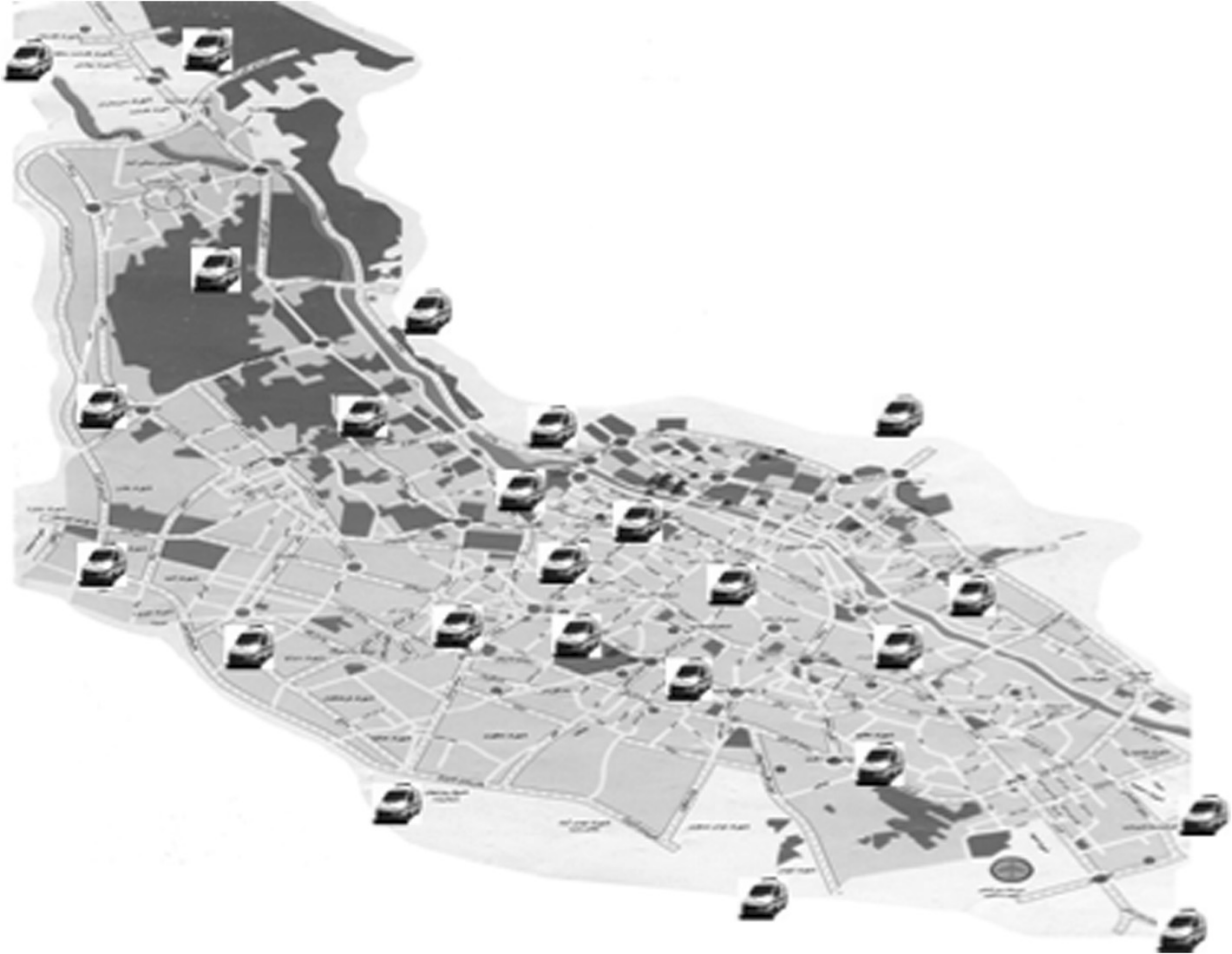
Position of permanent ambulance stations (PS). A total number of 24 ambulances are located in their permanent locations

**Fig. 2:**
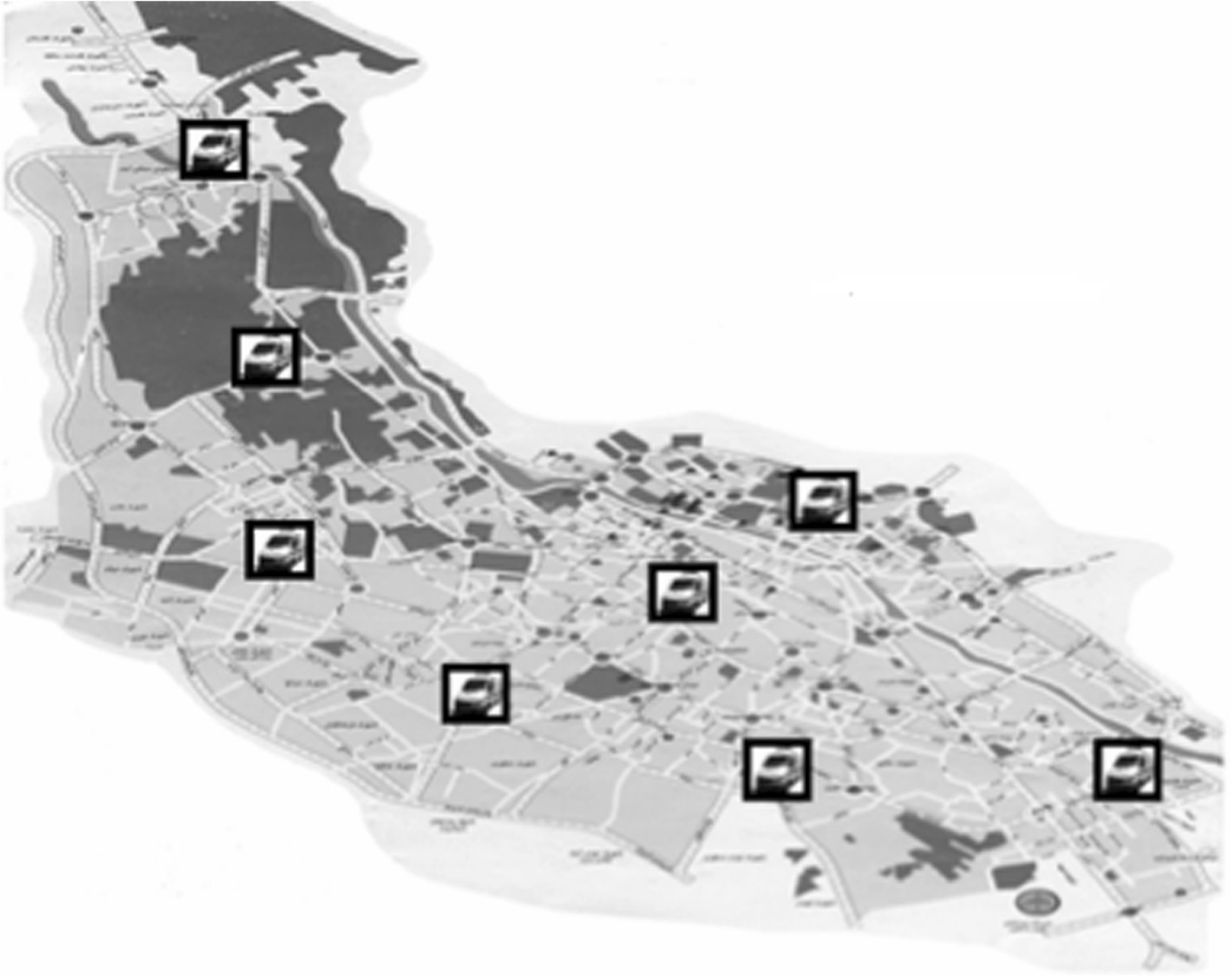
Position of temporary ambulance stations (TS). Eight ambulances work as temporary and rotating ambulances located in new areas

**Fig. 3:**
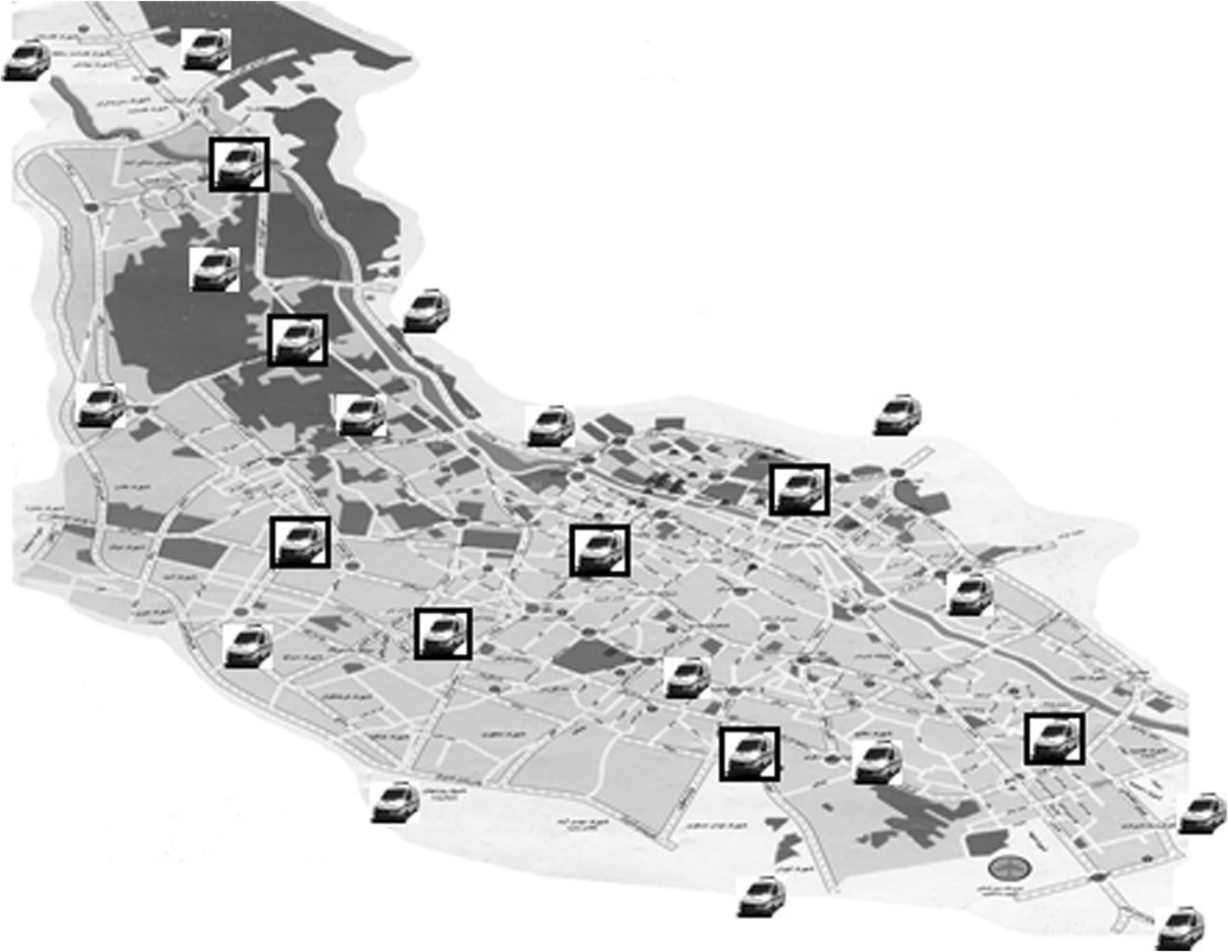
Position of permanent and temporary ambulance stations (PS and TS). Using the same resources, all 24 ambulances are located in 16 permanent locations and 8 temporary locations

The task of EMS is to rapidly and appropriately respond to medical emergencies [7–11]. Response times can be reduced by either increasing the number of available ambulances within a system or reducing the distance between the ambulance and the patient. However, the present financial situation does not allow expansion of the ambulance fleet and the crews needed [11]. Furthermore, the increasing number of inhabitants in an urban area will challenge the resources already in place. The traffic density in many metropolitan areas of the world causes situations sometimes referred to as “traffic infarction”, i.e., when traffic has more or less come to a complete stop due to congestion. In such situations, blue lights and sirens are of little value. Thus, alternative ways to improve response times are needed. Helicopter EMS (HEMS), as used in London, is hardly an option for LMIN due to the cost [11]. Thus, the concept of reducing the response times by temporarily deploying ambulances closer to the patients seems like a possible solution. Ideally, the temporary locations are chosen based on risk analysis and statistics on previous ambulance runs [12, 13].

Such interventions (“Fluid deployment”) can be limited to specific times (i.e., “peak hours”) and geographical areas and have been suggested and implemented in different EMS systems [13–15]. However, very little has been published regarding this subject, and to the best of our knowledge, the outcome in terms of mortality and morbidity has never been reported. Improving outcomes will be important for EMS structure, especially in LMIN countries where resources are limited.

Shiraz is the capital of the Fars province in Iran with a 10,531-sq. km area and 1.7 million inhabitants. Iranian EMS was established simultaneously in Iranian major cities in 1975. It was organized as a governmental organization with free-of-charge services for all patients. Since then, Iranian EMS has expanded and become a nationwide organization. However, this service has faced infrastructural problems such as traffic congestion, narrow roads, as well as lack of human resources and shortcomings in command and control systems; all are factors that have been observed in many other developing countries [2, 15]. During the study period, Shiraz EMS had 31 ambulances around the clock; 24 were stationed in the city and 7 in the rural area.

Each ambulance is staffed with one nurse with training in anesthesiology and one emergency medical technician (EMT) trained in basic life support (6 months training). Moreover, one ambulance in Shiraz is designed to work as a mobile intensive care unit (ICU), staffed with a team of one nurse anesthesiologist, one general practitioner (GP), and one EMT. Another physician works as consultant for the dispatchers to make medical decisions in difficult cases or when patients are suggested to be left at home. Besides national EMS, some private ambulance companies transfer non-urgent patients within this geographical area.

### Objectives

The aim of this study was to evaluate the Shiraz EMS response times and outcome (i.e., during on-scene treatment and transportation to the hospital; hospital mortality is not included) of patients in two groups, attended by permanently (PS) and temporarily (TS) stationed ambulances (“fluid deployment”).

## Methods

### Data collection

This study was conducted during two different time periods. Due to the large number of missions performed by PS ambulances vs. TS ambulances, a second pilot study was conducted to eliminate the potential bias in our statistical evaluation (see Methods and Results).

1. The first study was undertaken between March 21, 2012 and March 20, 2013. Two groups of patients were studied: those attended by crews dispatched from PS ambulances vs. ambulances temporarily deployed (TS) to specific locations in Shiraz. Normally, all 24 available ambulances were running as PS(Fig.1). However, during the selected time period, 8 out of 24 ambulances were located at temporary stations (TS)(Fig. 2). The temporary locations were chosen based on accumulated EMS data (EMS center’s registry), in which most geographically affected areas with regard to number of incidents and traffic congestion together with the distance to the nearest hospital were used to identify most appropriate locations for TS. The time, in which ambulances were temporarily deployed, was between 1–2 h every day (average 1.5 h), all weekdays, and at eight stations, simultaneously (1.5 h × 365 days = 547.5 h = 22.81 days). During this time, 16 remaining ambulances were running as other days with all ambulances based at permanent ambulance stations scattered around the city; they were, however, possible to dispatch by radio from any given location. The critical key factors were to compare response times and the outcome in patients. Response time was calculated based on consumed time from receiving the alarm call until ambulance arrival at the scene. Scene time was accounted as the time spent by the ambulance crew on the scene, and evacuation time was the time registered for transportation of a patient to a hospital. The outcome is defined as survival to the hospital’s emergency department (ED).
  The following parameters were studied: age, gender, and the cause of missions, response time, scene time, evacuation time, and total time for each mission. Survival to hospital between TS and PS was used to evaluate the outcome.
2. A new prospective study was conducted between January 17, 2015 and February 10, 2015 (24 days). Twenty-Four available ambulances were divided into three groups: A, B, C. Each group of ambulances (no = 8) was stationed as TS during an 8-day period, while the remaining two groups acted as PS ambulances(Fig. 3). Thus, all ambulances acted once as TS and twice as PS. All ambulances were equipped and staffed similarly. The missions were performed at designated peak time, between 10:00–12:00 a.m. each day.

### Ethical permission

This study was approved by the ethical committee of Shiraz Medical University (2011-100/7 Feb.2011).

### Statistical analysis

The following methods were used in this paper to statistically analyze the results:

1. One-sample proportional test to examine whether a sample value differs from a population value [17].
2. The One Sample Kolmogorov-Smirnov (K-S) test to examine whether data is normally distributed comes from a uniform, Poisson, or exponential distribution [17].
3. One-sample Wilcoxon test to examine the mean or median of a single population [17].
4. Two-sample test for equality of proportions [17].
5. Mann-Whitney-Wilcoxon Test to examine non-normal distributions [17].
6. Bootstrapping is a nonparametric method, which lets us compare estimated standard errors, confidence intervals, and hypothesis testing [18–23].

A more detailed description of the statistical analysis using Bootstrapping is found in Appendix A.

Statistical analysis was performed using the R, version 3.0.1. (https://www.r-project.org). *P* values less than 0.05 were considered as significant.

Quantitative variables were reported as mean ± SD; median, and qualitative data were reported in terms of proportions. For analytic purposes, the Mann-Whitney *U* test was used to compare the response times between temporary and permanently stationed ambulances. Moreover, a 2-sample test for equality of proportions was utilized to compare the mortality rate between the two groups of patients. Finally, logistic regression was used to report odds ratios (ORs). In this regression, response was a binary variable (response time ≤ 8 min vs. > 8 min; dead vs. alive), and the reference category was temporary stations.

## Results

### Study period 1

During the study period (2012–2013) Shiraz’s EMS performed 83,673 missions. A total number of 2132 missions were excluded due to insufficient data (addresses and localization). Of the remaining missions (81,541), 1571 (2 %), and 79,970 (98 %) were performed by TS and PS, respectively. Both groups were matched regarding distribution of gender (sig = 0.189) by 2-sample test for equality of proportions and age by Mann-Whitney-Wilcoxon Test (sig = 0.621).

Patient’s characteristics are displayed in Table 1. Around 61 % of patients were male, and 39 % were female. The most common age group (39.5 %) was between 21 and 40 years of age. This table also shows the number of missions performed by TS and PS. Approximately 95 % of missions were performed in urban areas and the other 5 % were in rural areas.

**Table 1:** Patients transported by Shiraz emergency medical services during study period 2012–2013

	Indicator		Number	Percent
Missions	Missions		81,541	100 %
	Missions by permanent stations (PS)		79,970	98 %
	Missions by temporary stations (TS)		1571	2 %
	Sex	Male	52,051	61 %
		Female	29,490	39 %
Patient characteristics	Age	Age group 1–10	1735	2.7 %
		Age group 10–20	7250	11.2 %
		Age group 21–40	25,565	39.5 %
		Age group 41–60	15,668	24.2 %
		Age group 61–up	14,509	22.4 %
		Total	64,727	100 %
		Mean ± SD	42.59 ± 21.49	
Locations	Urban		57,774	95 %
	Rural		3043	5 %

The causes of missions are presented in Table 2. These are grouped in different diagnosis categories and were similar in both groups. Trauma is the most common cause of dispatching an ambulance, followed by decreased level of consciousness, cardiovascular diseases, and neurological causes (including cerebrovascular diseases), respectively.

**Table 2:** The cause of dispatching an ambulance grouped in different disease categories

Reason for call	Temporary	%	Permanent	%
Trauma including orthopedic	684	43.68	32382	42.30
Decreased level of consciousness	195	12.45	11207	14.63
Cardiovascular	201	12.85	10699	13.97
Neurological including cerebrovascular	188	12.00	7748	10.12
Internal medicine including poisoning	155	9.90	6643	8.68
Respiratory	94	6.00	4967	6.48
Surgical include abdominal pain	37	2.36	1897	2.48
Psychological	7	0.45	546	0.71
Gynecology	5	0.31	486	0.63
Total	1566		76575	

In both groups of TS and PS ambulances, trauma is the most common cause of dispatching an ambulance, followed by neurological causes including cerebrovascular diseases and cardiovascular diseases

In order to evaluate the impact of different deployment of ambulances (TS vs PS), the outcomes were calculated statistically by looking at the response times and mortality rates. To avoid bias due to unequal sizes of our samples, three different evaluation approaches were used (see also Appendix A) [18–23].

Table 3 shows the time intervals in the two groups of ambulances. A 2-min reduction in response time in favor of TS was recorded (*P* < 0.001–[95 % CI, 1.975, 2.025]).

**Table 3:** Time Intervals registered during 2012–2013 between two groups of dispatched ambulances PS and TS

Variable	Minimum		Median		Maximum		Mean ± SD	
	PS	TS	PS	TS	PS	TS	PS	TS
Response time sec (min)	30	3	488 (8.13)	428 (7.13)	7267 (121.11)	2533 (42.21)	530 ± 258 (8.83 ± 4.3)	410 ± 358 (6.83 ± 5.13)
							*P* < 0.001	
Scene time sec (min)	30	5	950 (15.83)	972 (16.20)	8659 (144.31)	7804 (130.06)	1062 ± 754 (17.7 ± 12.56)	1057 ± 662 (17.61 ± 11.0)
Evacuation time sec (min)	0	0	1043 (17.38)	1177 (19.61)	7709 (128.48)	4816 (128.48)	1160 ± 666 (19.33 ± 11.1)	1246 ± 660 (20.76 ± 11)
Total time sec (min)	60	13	2166 (36.10)	2294 (38.23)	9999 (166.65)	9630 (160.50)	2491 ± 1668 (41.51 ± 27.8)	2520 ± 1692 (42 ± 28.2)

The numbers written in the first line are calculated in seconds and those given in the brackets are in minutes

Differences between the response times in two groups comparing missions in urban vs. rural areas based on pre-calculated 8 and 15 min response thresholds is presented in Table 4. Pre-calculated response thresholds are official figures set by the Iranian Department of Health and Welfare. For urban missions, results of logistic regression showed that the odds of the response time less than 8 min for PS vs. TS decreased by 14 % (OR = 0.86, *P* < 0.02) (Table 5). The rural missions were excluded from the analysis because of the few number of missions in this category.

**Table 4:** Response time registered during 2012–2013 between PS and TS dispatched from urban and rural stations, based on pre-calculated 8- and 15-ms response thresholds, respectively. These figures are official figures set by the department of health and welfare

Variable	Group	Urban			Rural		
Response time							
		≤8 min	>8 min	Total	≤15 min	>15 min	Total
	PS	30,246 (49 %)	31,887 (51 %)	62,133	456 (78 %)	132 (22 %)	597
	TS	668 (51 %)	635 (49 %)	1303	1 (100 %)	0 (0 %)	1
		*P* = 0.0258					
	Mean ± SD (min)	8.73 ± 4.18			11.16 ± 7.73

**Table 5:** Output logistic regression. In Urban missions, results of logistic regression showed that the odds of the response time less than 8 min for PS vs. TS decreased by 14 % (OR = 0.86, *P* < 0.02) (Table 5). The rural missions were excluded from the analysis because of the few number of missions in this category (*n* = 6)

		Coefficient	SE	OR	*P* value
Response time less than 8 min					
Station	Permanent	−0.1452	0.0652	0.86	0.0258
	Temporary	Ref			
Mortality					
Station	Permanent	0.4189	0.2043	1.52	0.0403
	Temporary	Ref			

Table 6 compares the outcome of patients transported by PS and TS. In general, only around 45 % of patients were transferred to the hospitals (44.6 % in PS compared to 44.8 % in TS). The remaining number of patients received definitive treatment at scene, i.e., were treated and left on place or sent home (36.2 % in PS vs. 35.1 % in TS) or were transported by other means or refused to be transported to the hospital. (10.3 % in PS vs. 11.2 % in TS).

**Table 6:** Outcome of missions between two groups of ambulances (PS = permanent, TS = temporary) 2012–2013

		PS	TS
		Number (percentage)	Number (percentage)
Results	Transfer to hospital	35,645 (44.6 %)	704 (44.8 %)
	Definitive treatment at scene	28,944 (36.2 %)	550 (35.1 %)
	Transfer with private vehicles/patient refusal/recovery	8217 (10.3 %)	176 (11.2 %)
	Dead before ambulance arrival	1884 (2.3 %)	25 (1.5 %)
	Died under treatment at scene	12	0
	Died during ambulance transport	15	0
	Total number of deaths and mortality rate	1911 (2.4 %)	25 (1.5 %)
		*P* = 0.04	
	Cancelation, wrong address, no injuries	5253 (6.6 %)	116 (7.4 %)
	Sum	79,970 (100 %)	1571 (100 %)

Approximately 45 % of patients in each group of ambulances were transferred to the hospital. The remaining (53.1 %) were patients who received definitive care at scene (36 %, the same in both groups), transported by private means or refused to be transported to the hospitals (10.5 %, the same in both groups), those in whom EMS crews did not find any injuries or they could not be found due to wrong addresses (6.6 %, the same in both groups). Mortality rate for patients transported by TS ambulances was 1.5 % compared to the rate reported for the PS (2.3 %) (*P* = 0.04). These rates are being calculated after elimination of cancelations, wrong addresses, and no-injury cases. The logistic regression showed that the mortality odds of PS vs. TS increased by 52 % (OR = 1.52, *P* = 0.04)

Non-survivors were divided into two groups;

- “Dead on EMS arrival”: Response times related to patients who were dead on EMS arrival, assuming that shorter response times could have increased their chances of survival. The mortality in TS and PS groups were 25 out of 1571 (1.5 %) vs. 1884 out of 79,970 (2.3 %). The difference is statistically significant (*P* = 0.04–[95 % CI, 0.006, 0.012]).
- “Died during EMS treatment”, i.e., those who died at the scene or during transport after initiation of treatment. None of the patients treated in the TS group died after ambulance arrival, while 27 patients died in PS group.

### Study period 2

The lower number of missions performed by TS ambulances in study period 1 was due to chosen methodology and the time selection. However, the number of missions in average for any TS ambulance/h was 0.36 missions, which is 1571 missions divided into 8 ambulances for 1.5 h in 365 days (547.5 h) compared to that of the PS ambulance; 0.38/h (79,970 missions divided into 24 ambulances in 24 h and a year).

In order to eliminate the possible statistical bias made by the number of missions in the first study, a new study was conducted (see Method). The results show that 474 missions were conducted in the second time period, of which, 329 were conducted by PS and 145 by TS ambulances. There was no statistically significant difference between the cohorts in this study compared to the first study; the mean (± SD) of age was 45.08 (±22.85), and 58.2 % cases were male vs. 41.8 female. The mean (± SD) response time for PS ambulances in this study was 12.39 (±5.48) min vs. 10.36 (±5.65) min for TS ambulances. The 2-min difference in response time in favor of TS ambulances was statistically significant using the One-Sample Kolmogorov-Smirnov and Mann-Whitney Tests (*P* < 0.001). This result is in accordance with the results found in the first study.

There was no significant difference in mortality before arrival of ambulances between PS and TS in the second study (PS = 17 vs. TS = 5). Moreover, no death was registered under treatment at the scene or during transfer to the hospital in any of the groups (PS nor TS), respectively.

## Discussion

Patients’ outcome and survival in pre-hospital environment is influenced by a number of variables. One important factor for patients in critical condition is the EMS response time [24]. Although some studies could not find any association between response time and outcome due to the small sample size or focus on severe cases such as trauma [24–26], it is logical to assume that shorter response times are beneficial to reduce mortality [9, 27–35].

The aim of this study was to evaluate whether a reduction in response time is possible to achieve by using temporarily stationed ambulances (fluid deployment). The outcome was measured as early mortality. Even though the number of missions in the two study groups was highly unequal in the first study period, a significant reduction of response time (2 min), as well as mortality was observed using the Bootstrapping technique. This result was further confirmed by analysis of study period 2, in which a lower and more equal number of missions were studied. The 2-min discrepancy in response time could also be shown in this study by using simpler statistics.

Additionally, fewer patients were dead on EMS arrival in the group of patients attended by TS ambulances (1.5 %) as compared to the other group (2.3 %) (*P* < 0.05). Since the call-takers dispatched an ambulance based on their medical index/interview criteria, which is based on ABCD, it seems logical to assume that patients were alive when the calls were received. Thus, since the patients in both groups were matched by diagnosis, age and gender, a shorter response time might increase the chances of early survival. There was neither any death registered in study period 2 after ambulance arrival/during transfer of patients to the hospital nor any significant discrepancy in deaths before ambulance arrival to the scene between PS and TS compared to study period 1. This might be due to the small sample size or the shorter period of the second study.

In this study, localization of the temporary stations was based on the calculations from the number of missions in that specific area or the geographical location by EMS staff. However, a future study might be needed to find new ways of approaching this selection by using digital devices, statistical analysis, or simulations [14, 36–39].

## Limitations

A limitation to this study is the absence of a 30-day follow-up for all patients. However, the primary goal of this study was to evaluate the mortality rate of the pre-hospital part and not in the hospital or post-hospital phases. We had a real attempt to study both 24-h mortality and at-discharge mortality and morbidity at all hospitals involved in this study. Unfortunately, the registries at all hospitals were not reliable, missing data was too much, and no real conclusions could be made. Therefore, we decided to study the mortality on scene and during transportation as the outcome indicators.

Another issue to consider in the first study was the fact that we have not measured the response time for PS ambulances, which were running simultaneously with TS ambulances. Although ambulance response times will be influenced by the actual distance to the patient when the ambulance is being dispatched, measuring the mean time (± SD), the times between the two groups of ambulances can be compared. However, a possible increase in response times for the PS ambulances, when running the systems in parallel, will obviously not be detected. In study 2, the response time for both groups was measured simultaneously, and the mean 2-min discrepancy between two groups in study two may indicate no raise in PS ambulances response time while using TS ambulances at the same time. The average number of missions was also the same in both groups, which also may indicate no overloading of PS ambulances.

An important point in this report is the amount of people dismissed from the scene either on their own or following the assessment of an ambulance crew. Since there is no follow-up for this group, the rate of morbidity or mortality associated with decisions made by ambulance crews could not be calculated or assessed. This issue should be addressed in a future study.

The highly unequal distribution of missions between PS and TS has been addressed by applying a specific statistical method (Appendix A) and by conducting the study 2.

## Conclusions

The deployment of temporarily stationed ambulances (fluid deployment) decreased the response times and may improve early survival in patients with life-threatening emergencies. The results of this study might have a global impact in the management of EMS systems.

## Appendix A

### Detailed statistical analysis

#### First approach

In this approach we used one-sample proportional test. This test will determine whether there is any difference in mortality rate between those transported by PS (the population, control group) vs. TS. A *P* value less than 0.05 is significant. The result showed a difference in mortality rate in favor of TS (*P* = 0.0468, % mortality 0.159343, 95 % CI, 0.01054429–0.02376338).

Furthermore, by using one-sample Wilcoxon test, we investigated whether there is any difference between mean response time in the population (PS) and the temporarily stationed ambulances. The result shows statistical difference in response time between TS and control population (PS) (*P* < 0.001).

#### Second and third approaches

In these approaches, the 2-sample test for equality of proportions has been used to compare mortality rate and the Mann-Whitney-Wilcoxon Test, to compare response time (the distribution of response time is not normal). Also, the 95 % Bootstrap percentile confidence interval was reported for the interest parameter as a way of using the data collected for a single experiment to simulate what the results might be if the experiment was repeated over and over with a new sample of subjects. The following steps were taken:

Step 1: obtaining a Bootstrap data set

- Sample *n* observations randomly with replacement from the temporarily stationed ambulances
- Sample *n* observations randomly with replacement from the permanently stationed ambulances

Step 2: Calculate the Bootstrap version of the statistic of interest (difference of mortality rate and response time)

Step 3: Repeat steps 1 and 2 1000 times to generate the Bootstrap distribution of an interest statistic (difference of mortality rate and response time)

Step 4: Report a 95 % Bootstrap percentile confidence interval for the interest parameter. The interval between the 2.5th and 97.5th percentiles of the Bootstrap distribution of a statistic is reported as a 95 % Bootstrap percentile confidence interval for the interest parameter (difference of mortality rate and response time).

Step 5: The test is significant if the 95 % Bootstrap percentile confidence interval for the interest parameter does not include the zero.

### Second approach

*Mortality:* First, we assumed that a 1.5 % decrease in the mortality rates is valuable in our samples (1.5 % of 81,000 missions, i.e., around 1200 lives per year) by deploying ambulances as TSA. The minimum sample size (by considering *α* = 0.05, *β* = 0.2, *z*_1-*α*/2_ = 1.96, *z*_1−*β*_ = 0.84) will be 1200 cases in each group. Thus, 1200 cases were randomly selected within each group. For more accuracy, 95 % confidence intervals based on the Bootstrap method was used [14–16]. This protocol has been done when the control groups were two and three times larger than the sample group. The results are shown in the following table, calculating mortality rate in two groups.

**Table 7:** ᅟ

	*n*	Proportion of dead	SD	Difference proportion	*P*	95 % Bootstrap percentile confidence interval for difference of proportion	
Temporary	1200	0.0158	1.30e–05	0.0108	0.09	(−0.0008, 0.0234)	0.04
Permanent	1200	0.0267	2.16e–05				
Temporary	950	0.0158	1.63e–05	0.0122	0.06	(0.0011, 0.0238)	0.02
Permanent	1895	0.0280	1.43e–05				
Temporary	860	0.0139	1.60e–05	0.0121	0.05	(0.0016, 0.0221)	0.01
Permanent	2575	0.0260	9.84e–06				

The gray rows of the above table show that resampling 1000 times with replacement from two groups results in less mortality rate in the TS compared to that of the PSA, and the 95 % Bootstrap percentile confidence interval for the difference of the proportions does not include the zero. The white rows of the above table, however, show that the 95 % Bootstrap percentile confidence interval for the difference of the proportions includes the zero, and the mortality rate in the TS, tends to, but is not significantly less than that of the PS in 40 times (from resampling 1000 times). This means that the mortality rate will significantly differ if a large number of patients is involved.

**Table 8:** ᅟ

		Response time					95 % Bootstrap percentile confidence interval for difference of means
Stationed	*n*	Median	This mean	SD	Mean difference	*P*	
Temporary	1200	381.5	410.5	361.87	116.19	<0.001	(90.05, 141.65)
Permanent	1200	481.5	526.7	242.35			
Temporary	950	384.50	402.50	352.04	128.90	<0.001	(102.05, 155.46)
Permanent	1895	491.00	531.30				
Temporary	860	381.5	398.8	358.54	127.14	<0.001	(101.03, 152.69)
Permanent	2575	489.0	526.0	226.54			

*Response time:* The results of the Bootstrap calculating response time in two groups.

The table below shows that resampling 1000 times with replacement from two groups results in significantly less response time in the TS than the PS, and the 95 % Bootstrap percentile confidence interval for the difference of the mean response time between two groups does not include the zero.

### Third approach

Based on the results, the reduction of the means of response time and mortality rate at the scene of incident was significant. To enhance the accuracy of our result, we generated 1000 Bootstrap samples without replacement in size of 1571 from permanently stationed ambulances [because of 1571 TS]. We reported descriptive statistics and 95 % Bootstrap percentile confidence interval for the interest parameters [19]. The results of the Bootstrap calculating mortality rate in two groups are as follows:

**Table 10:** ᅟ

	*N*	Proportion of dead	SD	Difference proportion	Sig	95 % Bootstrap percentile confidence interval for difference of proportion	
Temporary	1571	0.0159	9.96e–06	0.0115	0.04	(0.0006, 0.0146)	0.02
Permanent	1571	0.0274	1.70e–05				

The table above shows that resampling 1000 times without replacement from the permanently stationed ambulances results in significantly less mortality rate in the temporarily stationed ambulances vs. the permanently stationed ambulances, and the 95 % Bootstrap percentile confidence interval for the difference of the mortality rate between two groups does not include the zero. The results of the Bootstrap calculating response time in two groups are as follows:

**Table 9:** ᅟ

		Response time				Sig	95 % Bootstrap percentile confidence interval for difference of means
	*n*	Median	Mean	SD	Mean difference		
Temporary	1571	416.0	431.0	353.80	107.19	<0.001	(83.782, 114.159)
Permanent	1571	494.0	538.2	247.8			

The table above shows that resampling 1000 times without replacement from the permanently stationed ambulances results in a significantly lower mean response time in the temporarily stationed ambulances vs. the permanently stationed ambulances, and the 95 % Bootstrap percentile confidence interval for the difference of the mean response time between two groups does not include the zero.

## References

[1] Al-Shaqsi S, Al-Kashmiri A, Al-Hajri H, Al-Harthy A. Emergency medical services versus private transport of trauma patients in the Sultanate of Oman: a retrospective audit at the Sultan Qaboos University Hospital. Emerg Med J (2013): emermed-2013. 10.1136/emermed-2013-202779 23825061

[2] Peyravi M, Ortenwal P, Djalali A, Khorram-Manesh A (2013). An overview of shiraz emergency medical services, dispatch to treatment. Iranian Red Crescent Med J.

[3] Demetriades D, Chan L, Cornwell E, Belzberg H, Berne TV, Asensio J, Chan D, Eckstein M, Alo K (1996). Paramedic vs private transportation of trauma patients: effect on outcome. Arch Surg.

[4] Johnson NJ, Carr BG, Salhi R, Holena DN, Wolff C, Band RA (2013). Characteristics and outcomes of injured patients presenting by private vehicle in a state trauma system. Am J Emerg Med.

[5] Gache K, Couralet M, Nitenberg G, Leleu H, Minvielle E (2013). The role of calling EMS versus using private transportation in improving the management of stroke in France. Prehosp Emerg Care.

[6] Camerlingo M, Cesana BM, Tudose V, Simoncini G, Valoti O, Pozzi E, Zaninelli A, Ferrarese C (2013). Stroke-Unit and emergency medical service: a 48-month experience in northern Italy. Neurol Sci.

[7] Wallace SK, Abella BS, Shofer FS, Leary M, Agarwal AK, Mechem CC, Gaieski DF, Becker LB, Neumar RW, Band RA (2013). Effect of time of day on prehospital care and outcomes after out-of-hospital cardiac arrest. Circulation.

[8] Tanaka Y, Yamada H, Tamasaku S, Inaba H (2013). The fast emergency vehicle pre-emption system improved the outcomes of out-of-hospital cardiac arrest. Am J Emerg Med.

[9] Ohwaki K, Watanabe T, Shinohara T, Nakagomi T, Yano E (2013). Relationship between time from ambulance call to arrival at emergency center and level of consciousness at admission in severe stroke patients. Prehosp Disaster Med.

[10] Wilde ET (2013). Do emergency medical system response times matter for health outcomes?. Health Econ.

[11] Overton J (2002). Reimbursement in emergency medical services: how to adapt in a changing environment. Prehosp Emerg Care.

[12] Dean S (2004). The origins of system status management. Emerg Med Serv.

[13] Wu CH, Hwang KP (2009). Using a discrete*-*event simulation to balance ambulance availability and demand in static deployment systems. Acad Emerg Med.

[14] Feldman MJ, Lukins JL, Verbeek RP, MacDonald RD, Burgess RJ, Schwartz B (2004). Half-a-million strong: the emergency medical services response to a single-day, mass-gathering event. Prehosp Disaster Med.

[15] Branas CC, ReVelle CS, MacKenzie EJ (2000). A trauma resource allocation model for ambulances and hospitals. Health Serv Res.

[16] Peyravi MR, Tubaei F, Pourmohammadi K (2009). The efficiency of motorlance in comparison with ambulance in Shiraz, southern Iran. Iranian Red Crescent Med J.

[17] Sprent P, Nigel C. Applied nonparametric statistical methods. CRC Press, 2007.

[18] Singh, Kesar, and Minge Xie. "Bootstrap: a statistical method." Unpublished manuscript, Rutgers University, USA. Retrieved from http://www.stat.rutgers.edu/home/mxie/RCPapers/bootstrap.pdf (2008).

[19] R Library: Introduction to bootstrapping Available at: http://www.ats.ucla.edu/stat/r/library/bootstrap.htm.

[20] Mooney, Christopher Z., Robert D. Duval, and Robert Duval. Bootstrapping: A nonparametric approach to statistical inference. No. 94–95. Sage, 1993.

[21] Carpenter J, Bithell J (2000). Bootstrap confidence intervals: when, which, what? A practical guide for medical statisticians. Stat Med.

[22] Kotrlik JWKJW, Higgins CCHCC (2001). Organizational research: Determining appropriate sample size in survey research appropriate sample size in survey research. Inf Technol Learn Perform J.

[23] Davison AC, Diego K (2002). An Introduction to the Bootstrap with Applications in R. Statisticial Computing and Statistical Graphics Newsletter.

[24] Do HQ, Nielsen SL, Rasmussen LS (2010). Response interval is important for survival until admission after prehospital cardiac arrest. Dan Med Bull.

[25] Weiss S, Fullerton L, Oglesbee S, Duerden B, Froman P (2013). Does ambulance response time influence patient condition among patients with specific medical and trauma emergencies?. South Med J.

[26] Boehm KM (2013). “Commentary on” Does ambulance response time influence patient condition among patients with specific medical and trauma emergencies?. South Med J.

[27] Herlitz J, Bång A, Gunnarsson J, Engdahl J, Karlson BW, Lindqvist J, Waagstein L (2003). Factors associated with survival to hospital discharge among patients hospitalised alive after out of hospital cardiac arrest: change in outcome over 20 years in the community of Göteborg, Sweden. Heart.

[28] O’Keeffe C, Nicholl J, Turner J, Goodacre S. Role of ambulance response times in the survival of patients with out-of-hospital cardiac arrest. Emerg Med J (2010): emj-2009. 10.1136/emj.2009.086363 20798090

[29] Pell JP, Sirel JM, Marsden AK, Ford I, Cobbe SM (2001). Effect of reducing ambulance response times on deaths from out of hospital cardiac arrest: cohort study. BMJ.

[30] Kuo CW, See LC, Tu HT, Chen JC (2014). Adult out-of-hospital cardiac arrest based on chain of survival in Taoyuan County, northern Taiwan. J Emerg Med.

[31] Blackwell TH, Kaufman JS (2002). Response time effectiveness: comparison of response time and survival in an urban emergency medical services system. Acad Emerg Med.

[32] Gonzalez RP, Cummings GR, Phelan HA, Mulekar MS, Rodning CB (2009). Does increased emergency medical services prehospital time affect patient mortality in rural motor vehicle crashes? A statewide analysis. Am J Surg.

[33] Meng Q, Weng J (2013). Uncertainty analysis of accident notification time and emergency medical service response time in work zone traffic accidents. Traffic Inj Prev.

[34] Goh ES, Liang B, Fook-Chong S, Shahidah N, Soon SS, Yap S, Leong B (2013). Effect of location of out-of-hospital cardiac arrest on survival outcomes. Ann Acad Med Singapore.

[35] Biddinger PD, Baggish A, Harrington L, D’Hemecourt P, Hooley J, Jones J, Kue R, Troyanos C, Dyer KS (2013). Be prepared—the Boston Marathon and mass-casualty events. N Engl J Med.

[36] Rajagopalan HK, Saydam C, Xiao J (2008). A multiperiod set covering location model for dynamic redeployment of ambulances. Comput Oper Res.

[37] Azizan MH, Lim CS, Hatta WALWM, Go TL, Teoh SS. Simulation of Emergency Medical Services Delivery Performance Based on Real Map. International Journal of Engineering and Technology .Vol 5, no. 3, 2013:2620–2627.

[38] Branas CC, Wolff CS, Williams J, Margolis G, Carr BG (2013). Simulating changes to emergency care resources to compare system effectiveness. J Clin Epidemiol.

[39] Ong ME, Ng FS, Overton J, Yap S, Andresen D, Yong DK, Lim SH, Anantharaman V (2009). Geographic-time distribution of ambulance calls in Singapore: utility of geographic information system in ambulance deployment (CARE 3). Ann Acad Med Singapore.

